# New Appliances and Things Medical

**Published:** 1896-12-19

**Authors:** 


### NEW APPLIANCES AND THINGS MEDICAL.

**[We shall be glad to receive, at our Office, 28 & 29, Southampton Street, Strand, London, W.C., from the manufacturers, specimens of all new preparations and appliances which may be brought out from time to time.]**

### SOLUBLE COCOA.

(Ph. Suchard, Neuchaell, Paris, London, &c.)

This cocoa, as evidenced by the analysis of the great German chemists, Fresenius and Bischoff, is of the purest quality, made in a rational manner from good cocoa nibs, and with no trace of shell. It is highly soluble, and of delicate flavour, and, in fact, for household purposes there is no better or more convenient variety in the market.

### TABLE JELLIES.

(S. Chivers and Sons, Histon, Cambridge.)

We have received samples of the above well-known table jellies. Being carefully made, and containing only the purest of ingredients, we can thoroughly recommend them to the notice of our readers, and for invalids they may safely be relied upon as being no less valuable, from a diatetic and nutritional point of view, than the greatly-valued home-made calf's-foot jelly, over which they have the additional advantage of being made with one tithe of the trouble. A little hot water and a mould is all that is necessary, with the aid of one of these prepared packets, to make one pint of the very best table jelly that the most exacting invalid can require. As Messrs. Chivers and Sons' list shows, these jellies may be obtained in a large variety of flavouring.

### LIQUEUR WHISKY.

(W. Stenhouse and Co., Glasgow.)

We have had occasion to speak on a previous occasion in praise of this whisky, and are glad to call attention at this festive season to it as really wholesome and pure, and as one that may be advantageously chosen for medical and household purposes.

### HUGON'S REFINED BEEF SUET.

(Hugon and Co., Manchester.)

This preparation consists of pure compressed beef suet. It is prepared in a useful form, consisting of a firm block. It is admirably adapted for frying purposes in the place of lard, to which, if on account of its keeping properties alone, it is greatly superior. It is a most useful addition to the household.

